# Five ETS family members, ELF-1, ETV-4, ETV-3L, ETS-1, and ETS-2 upregulate human leukocyte-associated immunoglobulin-like receptor-1 gene basic promoter activity

**DOI:** 10.18632/aging.101475

**Published:** 2018-06-18

**Authors:** Qizhi Cao, Shude Yang, Qing Lv, Yan Liu, Li Li, Xiaojie Wu, Guiwu Qu, Xiaoli He, Xiaoshu Zhang, Shuqin Sun, Boqing Li, Jing An, Tao Hu, Jiangnan Xue

**Affiliations:** 1 Department of Immunology, School of Basic Medical Sciences, Binzhou Medical University, Shandong 264003, China; 2 Anti-aging Research Institution, Binzhou Medical University, Shandong 264003, China; 3 School of Agriculture, Ludong University, Shandong 264003, China; 4 Beijing Key Laboratory of Drug Target and Screening Research, Institute of Materia Medica, Chinese Academy of Medical Sciences and Peking Union Medical College, Beijing 100050, China; 5 School of Gerontology, Binzhou Medical University, Shandong 264003, China; 6 The People's Liberation Army 107 Hospital, Affiliated Hospital of Bin Zhou Medical University, Yantai 264002, China; 7 Department of Microbiology, School of Basic Medical Sciences, Binzhou Medical University, Shandong 264003, China; 8 School of Medicine, University of California - San Diego, La Jolla, CA 92037, USA; * Equal contribution

**Keywords:** LAIR-1, ETS transcription factors, promoter, ETS-2, transfection, Luciferase

## Abstract

Human leukocyte-associated immunoglobulin-like receptor-1 (LAIR-1), an immunoinhibitory receptor, is expressed on most types of hematopoietic cells and some tumor cells. LAIR-1 plays an inhibitory role in immune cell maturation, differentiation, and activation. LAIR-1 is also involved in some autoimmune diseases and tumors. However, the mechanism controlling the regulation of the LAIR-1 gene is still unknown. In order to elucidate the molecular mechanisms involved in LAIR-1 regulation, in the present study, we cloned and characterized the promoter region of LAIR-1 gene using a series of truncated promoter plasmids in luciferase reporter assays. Our results show that the basic core promoter of LAIR-1 is located within the region -256/-8 relative to the translational start site. Our further studies indicate that five ETS transcription factors: ELF-1, ETV-4, ETV-3L, ETS-1 and ETS-2, can up-regulate the LAIR-1 basic promoter activity. Of these, ETS-2 is the most effective transcription factor. Moreover, ETS-2 was confirmed to interact directly with the basic promoter of LAIR-1. This study presents the first description of regions/factors capable of up-regulation the promoter activity of LAIR-1. This new knowledge contributes to understanding of the molecular mechanisms involved in LAIR-1 associated immune regulation and diseases.

## Introduction

Human leukocyte-associated immunoglobulin-like receptor-1 (LAIR-1, also known as CD305), is an immunoinhibitory receptor for collagen and C1q complement component [1,2]. LAIR-1 is a 32-kDa transmembrane glycoprotein containing a single extracellular C2-type Ig-like domain and two immune receptor tyrosine-based inhibitory motifs (ITIMs) (VTYAQL and ITYAAV) in its cytoplasmic tail. LAIR-1 is structurally related to several other inhibitory immunoglobulin superfamily members, including human KIRs, human FcRa and immunoglobulin-like transcripts (ILTs), all of which are localized to the leukocyte receptor complex (LRC) on human chromosome 19q13.4 [3]. LAIR-1 is expressed by almost all immune cells, including NK cells, 70–80% CD4^+^T cells, 80–90% CD8^+^T cells, B cells, dendritic cells (DCs), monocytes and CD34^+^ hematopoietic progenitor cells. On peripheral blood mononuclear cells, LAIR-1 surface expression is greatly stimulated by treatment of the cells with PHA, while TGF-β and IL-15 treatment can reduce the PHA-induced up-regulation of LAIR-1expression [4]. TNF-α also can decrease the LAIR-1 expression in Th1 and Th2 cells [5]. However, the mechanism of LAIR-1 gene regulation is still unknown.

LAIR-1 plays an inhibitory role in immune cell maturation, differentiation, and activation, and regulates immune system balance [3,6,7]. Activation by a monoclonal anti-LAIR-1 antibody or engagement by collagen causes a phosphorylation of the tyrosine within LAIR-1 ITIMs and recruitment of the SH2-containing phosphatases SHP-1, SHP-2, or C-terminal Src kinase (Csk), which then negatively regulates intracellular signaling [6,8]. LAIR-1 can inhibit target cell lysis mediated by resting and activated NK cells and the cytotoxic activity of effector T cells upon CD3 cross-linking or antigen stimulation [3,9]. LAIR-1 also inhibits the differentiation of DCs from CD34^+^ peripheral blood cell precursors and megakaryocytes from primary human CD34^+^ hematopoietic progenitor cells cultured in the presence of cytokine cocktail plus thrombopoietin (TPO) [10,11]. C1q, one ligand for LAIR-1, can bind to LAIR-1 and lead to monocyte-to-DC maturation arrest and suppression of cytokine production by DCs [2]. Moreover, LAIR-1 has been involved in immune-related diseases and tumors. Expression of LAIR-1 is significantly decreased in the circulating CD4^+^ T cells in patients with rheumatoid arthritis [5] or leukemia [12]. LAIR-1 can inhibit GM-CSF-dependent proliferation in primary leukemia, prevent proliferation and induce apoptosis in human myeloid leukemia cell lines [13,14]. We found that LAIR-1 is expressed in epithelial ovarian cancer cells and is involved in cell proliferation and invasion of the ovarian cancer cell line HO8910 [15].

In our previous study, we predicted LAIR-1 core promoter region using software Promoter 2.0, CorePromoter and Neural Network Promoter. We found a DNA sequence located within the region -600 to -200 bp from the translational start site (TSS) of LAIR-1 gene contains one TATA-box and one CAAT-box; within this region, there are several putative binding sites of transcription factors, such as ETS family [16]. ETS transcription factors have been implicated in the development, differentiation, and function of T cell, as well as the key drivers of leukemia and many solid tumors [17–23]. However, the detailed mechanism underlying LAIR-1 action and its relationship with ETS family members in normal and tumor cells is unclear. Exploration of the mechanism of LAIR-1 gene regulation will further the current understanding of the relationship of the LAIR-1 gene to the corresponding diseases. In this study, we have identified the basic core promoter of human LAIR-1 gene for the first time, and studied the regulation of this promoter by ETS family transcription factors, including ELF-1, ETV-4, ETV-3L, ETS-1, and ETS-2.

## RESULTS

### Determination of the minimal promoter region of LAIR-1

**Figure 1:**
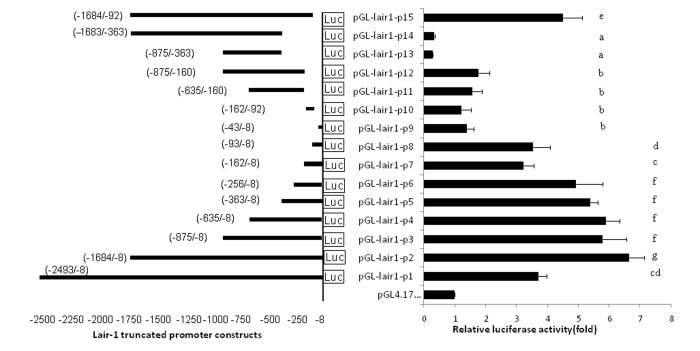
**The location and relative luciferase activities of LAIR-1 truncated promoter constructs.** The LAIR-1 gene promoter region and its 15 truncated constructs are presented on the left. The nucleotide positions of each DNA fragment in the constructs are indicated by numbers; the translational start site (TSS) is defined as the +1 position. Relative luciferase activities in HEK293T cells transfected with each truncated construct are presented on the right. Luciferase activities were measured at 48 h after transfection. The results are the mean ± standard deviation of triplicate transfections and are expressed in arbitrary units based on the fireﬂy luciferase activity normalized against the Renilla luciferase activity. The bars present as the mean ± standard deviation of three independent transfection experiments (a, b, c, d, e, f and g indicate significant differences in groups identified with 1-way ANOVA,p<0.05).

The minimum sequence required for promoter activity was determined by generating a series of luciferase reporter plasmids containing truncated DNA fragments of the LAIR-1 promoter sequence and transiently transfecting into HEK293T cells. The promoter activity of these truncated DNA fragments was measured by luciferase assay (Fig. 1). The luciferase activity of pGL-lair1-p2 (-1684/-8) had relatively higher promoter activity and showed a 6.65-fold increase compared with the empty pGL4.17 vector, indicating a functional promoter in the -1684 to -8 region relative to TSS of LAIR-1 gene. When the DNA sequence was deleted to position -875, the promoter activity with pGL-lair1-p3 (-875/-8) decreased significantly compared with the pGL-lair1-p2 (-1684/-8) (Fig. 1), this result demonstrates that positive regulatory elements may be located in the -1684 to -875 region. 5’ deletions from position −875 to -256 did not significantly change activity; A further deletion to -162 significantly decreased the promoter activity of pGL-lair1-p7 (-162/-8) when compared with pGL-lair1-p6 (-256/-8), indicating the presence of one positive regulatory element may be located in this deleted region. Analysis of luciferase activities of pGL-lair1-p7 (-162/-8), pGL-lair1-p8 (-93/-8), and pGL-lair1-p9 (-43/-8), demonstrates the possible presence of one positive regulatory element and one negative regulatory element at the -93 to -43, and -162 to -93 regions, respectively. These results indicate that pGL-lair1-p6 (-256/-8) has the basic promoter activity, that the minimal promoter of the LAIR-1 gene may be located at the -256 to -8 region relative to TSS, and that this region possibly contains one negative and two positive regulatory elements.

### Regulation of LAIR-1 basic core promoter by transcription factors ETS family

**Figure 2:**
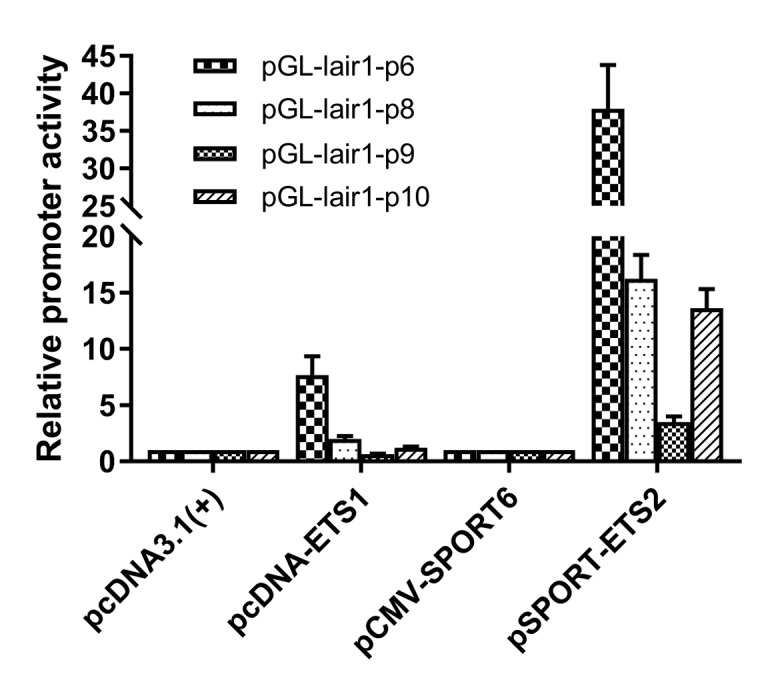
**Regulation of the LAIR-1 basic promoter by ETS-1 and ETS-2.** Expression vector of ETS-1 (pcDNA-ETS1) or ETS-2 (pSPORT-ETS2) was co-transfected into HEK297T cells with pGL-lair1-p6 (-256/-8), pGL-lair1-p8 (-93/-8), pGL-lair1-p9 (-43/-8), or pGL-lair1-p10 (-162/-92). Luciferase activities were measured at 48 h after transfection. The bars represent the mean ± standard deviation of three independent transfection experiments.

**Figure 3:**
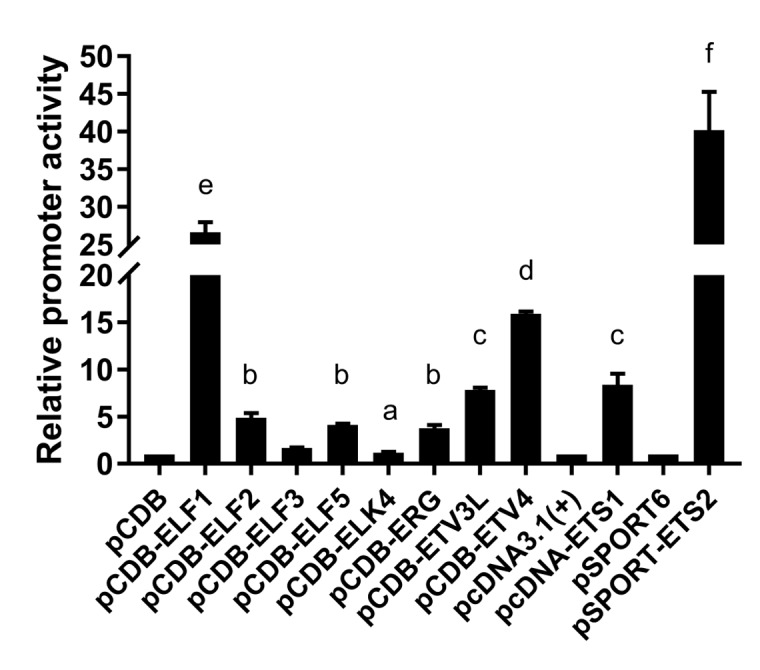
**Regulation of the basic LAIR-1 promoter activity by ETS transcription factors**. Expression vector of ELF-1 (pCDB-ELF1), ELF-2 (pCDB-ELF2), ELF-3 (pCDB-ELF3), ELF-5 (pCDB-ELF5), ELK-4 (pCDB-ELK4), ERG (pCDB-ERG), ETV-3L (pCDB-ETV3L), ETV-4 (pCDB-ETV4), ETS-1 (pcDNA-ETS1) or ETS-2 (pSPORT-ETS2) was co-transfected with pGL-lair1-p6 (-256/-8), into HEK297T cells. Luciferase activities were measured at 48 h after transfection. The bars represent the mean ± standard deviation of three independent transfection experiments (a, b, c, d, e, and f indicate significant differences in groups identified with 1-way ANOVA, p<0.05).

The putative transcription factor binding sites within the basic promoter of lair-1 sequence were identified using online PROMO software (http://alggen.lsi.upc.es/cgi-bin/ promo_v3/promo/promoinit.cgi?dirDB=TF_8.3) and JASPAR database (http://jaspar.genereg. net/). After we ranked them in descending order according to the relative scores, about 50% of the top 30 predicted transcription factors belonged to the ETS family, with the ETS-like, ELK-like and ELF-1-like subfamilies were dominating. Therefore, we chose several transcription factors from these three ETS subfamily (ETS-like subfamily: ETS-1, ETS-2, ERG and ETV3L; ELK-like subfamily: ELK-4 and ETV-4; ELF-1-like subfamily: ELF-1 and ELF-2.) to determine their potential effects on the regulation of the lair-1 promoter activity. We also chose two EHF-like family members (ELF-3 and ELF-5), which has been reported to be involved in ovarian cancer, to detect whether they can regulate LAIR promoter activity [24]. We first investigated whether ETS-1 and ETS-2 could regulate the minimal promoter activity of LAIR-1. We co-transfected with ETS-1 or ETS-2 expression vectors, and pGL-lair1-p6 (-256/-8), pGL-lair1-p8 (-93/-8), pGL-lair1-p9 (-43/-8) or pGL-lair1-p10 (-162/-92) into HEK293T cells, we found ETS-1 and ETS-2 increased pGL-lair1-p6 (-256/-8) activity to 7.67- and 37.95- fold, respectively. ETS-2, but not ETS-1, also increased the activity of pGL-lair1-p8 (-93/-8) and pGL-lair1-p10 (-162/-92) (Fig. 2). The results confirmed that -256/-8 had the basic promoter activity which can be regulated by ETS-1 and ETS-2. We further examined the effects of other ETS members including ELF-1, ELF-2, ELF-3, ELF-5, ELK-4, ERG, ETV-3L, and ETV-4, on the LAIR-1 basic promoter activity. As shown in Figure 3, ELF-1, ETV-4, ETV-3L, ETS-1 and ETS-2 significantly enhanced the basic promoter activity, and ETS-2 was the most effective transcription factor.

### Effect of ELF-1, ETV-4, ETV-3L, ETS-1 and ETS-2 on LAIR-1 gene expression in HO8910 cells

**Figure 4:**
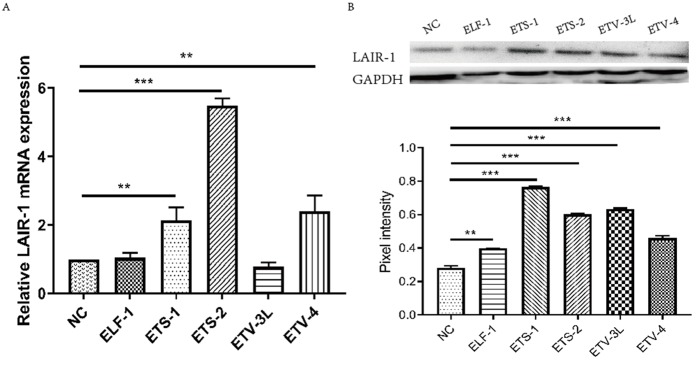
**Effect of ELF-1, ETV-4, ETV-3L, ETS-1 and ETS-2 on LAIR-1 gene expression in HO8910 cells.** HO8910 cells were transfected with pGL-lair1-p6 (-256/-8) and expression vector of ELF-1 (pCDB-ELF1), ETV-3L (pCDB-ETV3L), ETV-4 (pCDB-ETV4), ETS-1 (pcDNA-ETS1) or ETS-2 (pSPORT-ETS2). At 48 h post-transfection, cells were harvested. (**A**) Total RNA was used to detect the LAIR-1 mRNA level through qRT-PCR. The relative mRNA level was obtained after comparison with the empty vector, which was set to 1. (**B**) LAIR-1 protein expression was analyzed by Western blotting. The experiments were repeated at least thrice, and the data from one representative experiment with two technical repeats were presented (**, p < 0.01; ***, p < 0.001).

According the results of the luciferase assays, we know that ELF-1, ETV-4, ETV-3L, ETS-1 and ETS-2 can enhance LAIR-1 basic promoter activity. We further studied the effect of these transcription factors on the regulation of LAIR-1 gene expression in ovarian cancer cell HO8910, which we have used in our previous study to investigate the function of LAIR-1 [15]. As shown in Figure 4, five ETS members had distinctive regulation efficiency on LAIR-1 expression in HO8910. All five factors can enhance LAIR-1 protein expression, while ETS-1, ETS-2 and ETV-4 can significantly increase LAIR-1 mRNA expression; Consistent with the results of the luciferase assays, ETS-2 is the most efficient transcription factor, which can significantly increase the LAIR-1 mRNA expression in HO8910.

### Binding of ETS-2 to the LAIR-1 basic promoter

**Figure 5:**
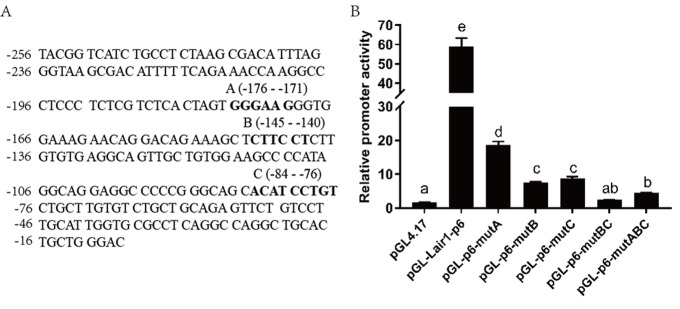
**Effect of ETS-2 on the pGL-lair1-p6 (-256/-8) and ETS binding site mutant constructs.** (**A**) DNA sequence of the LAIR-1 gene basic promoter (-256 to -8 relatives to the translational start site); The ETS binding sites A, B and C predicted by corresponding software are shown with bold-type letter. (**B**) Putative ETS binding site (**A**, **B** and **C**) deletion mutagenesis was carried out in the construct pGL-lair1-p6 (-256/-8). Expression vector of ETS-2 (pSPORT-ETS2) was co-transfected with the pGL-lair1-p6 (-256/-8) or other diﬀerent mutant constructs, into HEK293T cells. Luciferase activities were measured at 48 h after transfection. The bars present the mean ± standard deviation of three independent transfection experiments (a, b, c, d, and e indicate significant differences in groups identified with 1-way ANOVA, p<0.05).

Because ETS-2 can upregulate both LAIR-1 basic promoter activity and gene expression with high efficiency, suggesting there may be a direct interaction between ETS-2 and LAIR-1 basic promoter. Based on the analysis results obtained using online software PROMO and JASPAR database, three highly potential ETS-2 binding sites within the LAIR-1 basic promoter, A-site (5’-GGGAAT-3’, at position -176 to -171), B-site (5’-CTTCCT-3’, at position -145 to -140) and C-site (5’-ACATCCTGT-3’, at position -84 to -76), were identified (Fig 5A). In order to investigate the importance of these three sites in LAIR-1 basic promoter activity, we constructed a series of DNA report plasmids with deletion mutations of these sites and co-transfected HEK293T cells with these plasmids and ETS-2 expression vector. As shown in Figure 5B, the LAIR-1 promoter activity of pGL-lair1-p6, which has all sites intact, was increased by nearly 60-fold when compared with the basic pGL4.17. Disruption of either site significantly reduced the activity. These results indicate that all three ETS-2 binding sites are necessary for maximal promoter activity; however, the A-site is least effective, while the B-site and C-site are the most effective.

**Figure 6:**
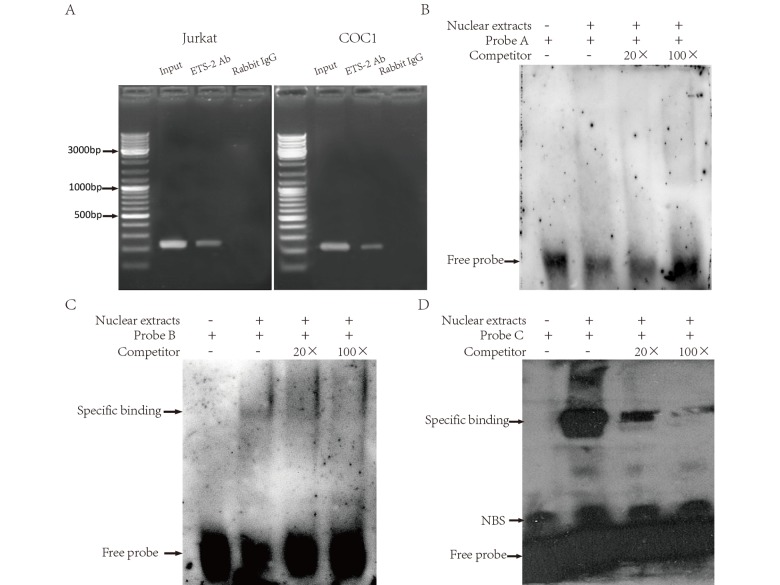
**Binding of ETS-2 to the human LAIR-1 gene basic promoter.** (**A**) CHIP assays. Chromatins from Jurkat (Left) and COC1 cells (Right) were prepared. Shown are the PCR products from: Lane 1, Chromatin before immunoprecipitation (input chromatin); Lane 2, Chromatin incubated with rabbit anti-ETS-2 antibodies; Lane 3, Chromatin incubated with negative control (normal rabbit IgG). (**B**, **C** and **D**) Electrophoretic mobility shift assays (EMSA). Nuclear extracts from Hela cells were subjected to EMSA. The use of biotin-labeled oligonucleotide probes **A**, **B** and **C** carrying the ETS binding site **A**, **B** and **C**, respectively, allowed assessment of the specificity of the protein-DNA binding. Competitors were unlabeled oligonucleotide probes, supplied at 20-fold and 100-fold concentrations. Specific binding: protein-DNA complexes of proteins and the probes. NSB: nonspecific binding. Free probe: free biotin-labeled oligonucleotide probes that did not bind to the proteins. The experiments were repeated at least three times.

We used CHIP assays to examine in vivo the association of ETS-2 with the LAIR-1 gene basic promoter region. Jurkat and COC1 cells were used because of their high expression of LAIR-1. As shown in Figure 6A, the PCR product obtained with CHIP DNA using the -256 to -8 region sequence primers indicated a specific interaction between ETS-2 and a chromatin fragment containing this promoter region. This interaction was observed both in Jurkat cells and COC1 cells. We further used EMSA assay to investigate whether ETS-2 could physically bind in vitro with the above three binding sites. As shown in Figure 6B-D, the nuclear protein from Hela cell expressing high level of ETS-2 bound to B-site and C-site (Fig. 6C, *lane* 2; Fig. 6D, *lane 2*), but not to the A-site (Fig. 5A, *lane* 2). Competition assays using an excess of unlabeled probe verified the specificity of both B-site and C-site probe/DNA interaction. EMSA results further confirmed both B and C-site are effective binding sites for ETS-2.

## DISCUSSION

We determined that a DNA sequence located in the -256 to -8 region relatives to the TSS of LAIR-1 gene has basic promoter activity, which can be regulated by ETS transcription factors ELF-1, ETV-4, ETV-3L, ETS-1 and ETS-2. ETS-2 shows the greatest enhancement of LAIR-1 promoter activity among these ETS factors. These results confirmed our previous computational prediction regarding the LAIR-1 promoter region [16]. Most importantly, we identified that ETS-2 specifically interacts with LAIR-1 promoter sequence in both Jurkat and COC1 cells; In vitro mutagenesis studies and EMSA assay further confirmed two ETS-2 binding sites within the basic promoter region are contribute to LAIR-1 promoter activity.

The ETS transcription factors comprise 28 members in humans, and these share a highly conserved ETS domain that recognizes the core (A/C)GGA(A/T) motif in the promoter or enhancer of the target genes [25]. The ETS proteins have been implicated in the development, differentiation and function of immune cells, as well as in the proliferation, migration, invasion, angiogenesis, and apoptosis of cancer cells from different tissues [20,21,25–28]. In the present study, in vivo screening of some candidate ETS proteins revealed that ELF-1, ETV-4, ETV-3L, ETS-1 and ETS-2 can enhance the LAIR-1 basic promoter activity with different efficiency, and the CHIP results confirmed that ETS-2 can bind to the LAIR-1 promoter sequence in Jurkat (an immortalized human T lymphocytes) and COC1 (one ovarian cancer cell lines) cells. We also found that the regulation effect of five transcription factor on expression of LAIR-1 gene expression in HO8910 cells is different. Above results indicate that different ETS factors may modulate the LAIR-1 in specific immune cells or tumor cells. This can be explained by the facts that the expression of ETS factors is cell-dependent, the different ETS factors show different tissue distribution and patterns [23]. For example, although ETS-1, ETS-2 and ELF-1 have the broadest range of tissue distribution, ETS-1 is highly expressed in spleen and thymus, which suggests the regulation of ETS-1 on the LAIR-1 expression maybe involved in the function of immune cells in the spleen and in the differentiation of T cells in thymus [3,28]. However, many ETS protein can be co-expressed in a single cell type, and form complex with other ETS proteins. ETV-4 has been reported to be associated with ETS-2 and ERG, and formed a complex integrated transcriptional network in PC3 cell nuclear extracts and prostate cancer tissues [21]. Our CHIP results of LAIR-1 promoter in Jurkat and COC1 cells only using anti ETS-2 antibody does not preclude the relevance of other ETS proteins [23]. Thus, the function of ETS factors in the regulation of LAIR-1 expression still needs further study for full elucidation.

Our previous study showed that LAIR-1 is highly expressed in COC1 and other ovarian cancer cells including HO8910, and its expression inhibits cell proliferation and invasion of the HO8910 cell line [15]. Our current results demonstrate ETS-2 binds directly to LAIR-1 promoter and positive regulates the expression of LAIR-1 in HO8910. ETS-2 is an important nuclear effector of the RAS/MAPK cascade, and it mediates the activation of gene expression programs downstream of RAS/MAPK signaling. Aberrant activation of RAS/MAPK signaling is a known driver of more than one third of all human carcinomas. The binding of ETS-2 to LAIR-1 promoter leads to high expression of LAIR-1, may suppress the development of ovarian cancer [15]. This is consistent with previous studies indicating ETS-2 is tumor suppressive [30]. However, previous studies reported that both ELF-1 and LAIR-1 expression were significantly associated with histological grade of ovarian carcinoma [15,31], while we overexpressed ELF-1 in HO8910 cells did increase LAIR-1 expression. Although the relationship between ELF-1, ETS-1,ETS-2 and LAIR-1 in ovarian carcinogenesis requires further investigation, it is possible that these studies may lead to development of therapies and prophylaxis in carcinogenesis.

In conclusion, we have identified the basic promoter region of the LAIR-1 gene and have demonstrated that the ETS transcription factors ELF-1, ETV-4, ETV-3L, ETS-1 and ETS-2 can up-regulate the basic promoter activity of this gene. Among these, ETS-2 is the most effective transcription factor which can bind directly to the basic promoter. These findings provide insights into the regulation of the LAIR-1 gene in immune cells, as well as some tumor cells. This new knowledge enhances the current understanding of the molecular mechanisms involved in the LAIR-1 associated immune regulation and diseases.

## MATERIALS AND METHODS

### Plasmid construction of truncated and deletion mutants of the LAIR-1 basic promoter

Based on our previous study bioinformatics analysis [16], we used a DNA sequence of 2485 bp(-2493 to -8)upstream from the translational start site (TSS) in the LAIR-1 gene as the candidate for identifying the promoter region. The TSS was defined as the +1 position. This 2485 bp fragment was amplified by PCR using genomic DNA extracted from human peripheral blood mononuclear cells and cloned into a pGL4.17-Luc luciferase reporter vector (Promega) using the restriction endonuclease KpnI and XhoI sites. The primer sequences were 5’-CGG GGTACC CCC CTG TCT GTC CAC GAG ATC CAA-3’ (underlined regions denote the KpnI recognition sequence sites) and 5’-TCC GCTCGAG GT CCC AGC AGT GCA GCC TGG-3’ (underlined regions denote XhoI recognition sequence sites). This report plasmid was designated as pGL-lair1-P1. A series of truncated fragments of the regulation region of the LAIR-1 gene were created by PCR amplification using the primers listed in Table 1 and pGL-lair1-P1 as template, and these were inserted into a pGL4.17-Luc luciferase reporter vector using the same restriction endonuclease Kpn I and Xho I sites. These plasmids containing the truncated fragments were designated as pGL-lair1-P2 to pGL-lair1-P15. Plasmids harboring deletion mutations within the core LAIR-1 promoter were constructed with a site-specific mutagenesis protocol using an overlap extension PCR and the primers listed in Table 2 [32]. DNA sequences of all the clones (the wild-type and mutated promoters) were verified by DNA sequencing. Expression vectors of transcription factor, ETS-1 (pcDNA-ETS1), ETS-2 (pSPORT-ETS2) ELF-1 (pCDB-ELF1), ELF-2 (pCDB-ELF2), ELF-3 (pCDB-ELF3), ELF-5 (pCDB-ELF5), ELK-4 (pCDB-ELK4), ERG (pCDB-ERG), ETV-3L (pCDB-ETV3L), ETV-4 (pCDB-ETV4) were purchased from Sino-GenoMax Company Limited (Beijing, China).

**Table 1:** Sequences of the primers used in the amplification of truncated fragments of the human LAIR-1 gene promoter.

Plasmid Name	Sequences of primer (5'-3')	Location
pGL-lair1-p2	5’-TCCG**CTCGAG**GTCCCAGCAGTGCAGCCTGG-3’ 5’-CGG**GGTACC**TCTTCCGCCAGACACCCTGG-3’	-1684 to -8
pGL-lair1-p3	5’-TCCG**CTCGAG**GTCCCAGCAGTGCAGCCTGG-3’ 5’-CGG**GGTACC**TTCCAAGTAGCTGTGCCCACC-3’	-875 to -8
pGL-lair1-p4	5’-TCCG**CTCGAG**GTCCCAGCAGTGCAGCCTGG-3’ 5’-CGG**GGTACC**AGGAACCTCCCCACTGTTCTC-3’	-635 to-8
pGL-lair1-p5	5’-TCCG**CTCGAG**GTCCCAGCAGTGCAGCCTGG-3’ 5’-CGG**GGTACC**CCGTTAACTTGTGGAGTTGGG-3’	-363 to -8
pGL-lair1-p6	5’-TCCG**CTCGAG**GTCCCAGCAGTGCAGCCTGG-3’ 5’-CCG**GGTACC**TACGGTCATCTGCCTCTAAGC-3’	-256 to -8
pGL-lair1-p7	5’-TCCG**CTCGAG**GTCCCAGCAGTGCAGCCTGG-3’ 5’-CGG**GGTACC**GAACAGGACAGAAAGCTCTTC-3’	-162 to -8
pGL-lair1-p8	5’-TCCG**CTCGAG**GTCCCAGCAGTGCAGCCTGG-3’ 5’-CCG**GGTACCC**GGGCAGCACATCCTGTCTG-3’	-93 to -8
pGL-lair1-p9	5’-TCCG**CTCGAG**GTCCCAGCAGTGCAGCCTGG-3’ 5’-CCG**GGTACC**ATTGGTGCGCCTCAGGCCAG-3’	-43 to -8
pGL-lair1-p10	5’-CCG**CTCGAG**CGGGGGCCTCCTGCCTATGG-3’ 5’-CGG**GGTACC**GAACAGGACAGAAAGCTCTTC-3’	-162 to -92
pGL-lair1-p11	5’-CCG**CTCGAG**TTCTTTCCACCCTTCCCACTA-3’ 5’-CGG**GGTACC**AGGAACCTCCCCACTGTTCTC-3’	-635 to -160
pGL-lair1-p12	5’-CCG**CTCGAG**TTCTTTCCACCCTTCCCACTA-3’ 5’-CGG**GGTACC**TTCCAAGTAGCTGTGCCCACC-3’	-875 to -160
pGL-lair1-p13	5’-CCG**CTCGAG**CGGTCGCAGCTCTTGGGCAA-3’ 5’-CGG**GGTACC**TTCCAAGTAGCTGTGCCCACC-3’	-875 to -263
pGL-lair1-p14	5’-CCG**CTCGAG**CGGTCGCAGCTCTTGGGCAA-3’ 5’-CGG**GGTACC**CTTCCGCCAGACACCCTGGC-3’	-1683 to -263
pGL-lair1-p15	5’-CCG**CTCGAG**CGGGGGCCTCCTGCCTATGG-3’	-1684 to -92
	5’- CGG**GGTACC**TCTTCCGCCAGACACCCTGG -3**’**	

The translational start site (TSS) in the LAIR-1 gene was defined as the +1 position; The underlined regions (CTCGAG/CCTACC) denote the XhoI / KpnI recognition sequence sites.

**Table 2:** Sequences of the primers used in deletion mutant construction of the human LAIR-1 gene promoter.

**mutant construction**	**primers**	**Sequences of primer (5'-3')**
pGL-p6-mutA	primer 1	5' -TAG CAA AAT AGG CTG TCC C- 3'
	primer 2	5'-TTTCCACCCACTAGTGAGACGAGAGGGAG-3'
	primer 3	5'- TCTCACTAGTGGGTGGAAAGAACAGGAC- 3'
	primer 4	5'- AAG CTG GAA GTC GAG CTT C-3'
pGL-p6-mutB	primer 1	5'- TAG CAA AAT AGG CTG TCC C-3'
	primer 2	5'-TGCCTCACACAACTTTCTGTCCTGTTCTTTC-3'
	primer 3	5'-CAGGACAGAAAGTTGTGTGAGGCAGTTGCTG -3'
	primer 4	5'-AAG CTG GAA GTC GAG CTT C-3'
pGL-p6-mutC	primer 1	5'-TAG CAA AAT AGG CTG TCC C-3'
	primer 2	5'- ACACAAGCAGACTGCTGCCCGGGGGCCTCC -3'
	primer 3	5'- CGGGCAGCAGTCTGCTTGTGTCTGCTGC- 3'
	primer 4	5'-AAG CTG GAA GTC GAG CTT C- 3'

### Transient cell transfection

Promoter activity was analyzed using a promoterless pGL4.17-basic vector as a negative control, and plasmid pRL-TK (Promega) was cotransfected as an internal control. All plasmids were propagated in *Escherichia coli* DH5α and isolated using a QIAprep spin miniprep kit (Qiagen). HEK293T cells were plated in 96-well plates 24 h before transfection, and triple wells were set for each group. A 100 ng of pGL4.17 vector containing different truncated DNA fragments and 4.0 ng amount of pRL-TK vector were cotransfected for each well using Vigofect (Vigofect Inc. Beijing, China), according to the manufacturer’s instructions. The regulation of the LAIR-1 promoter by ETS family members was investigated by cotransfecting an expression vector of transcript factors using Vigofect; the corresponding basic vector was used as a negative control.

### Luciferase assay

After 48 h of transfection, cell lysates were prepared for the measurement of luciferase activity using the Dual-Luciferase^®^ Reporter Assay System (Promega). The promoter activity was calculated from the chemical luminescence intensity ratio of firefly: Renilla luciferase for each construct; and then compared with the activity of vector pGL4.17 luciferase. The data were presented as the mean ± S.D from three independent experiments.

### Protein immunoblotting and RNA quantification

Effects of ELF-1, ETV-4, ETV-3L, ETS-1, and ETS-2 on LAIR-1 gene expression were analyzed using Quantitative real time PCR (qRT-PCR) and Western blotting analysis. An ovarian cancer cell line HO8910 was used in the assay. Cells were plated in 6-well plates 18 h before transfection, and triple wells were set for each group. 1μg transcription factor expression vector was transfected for each well using Vigofect (Vigofect Inc. Beijing, China), according to the manufacturer’s instructions. Cells were collected after 48 h transfection. Total RNA was extracted using Trizol reagent (Invitrogen, USA) and reverse-transcribed to cDNA using the Revert Aid First Strand cDNA Synthesis Kit (Fermentas, Canada). qRT-PCR was performed using Thermo Scientific DyNAmo ColorFlash SYBR Green qPCR kit (F-416). The forward and reverse primers used for lair-1 PCR amplification were 5'- TCT CCT CCT CCT GGT CCT CTT C-3' and 5'- GCC TTG TCT GCT GTC CTC TCT A-3'. Housekeeping gene GAPDH was used as the internal control, the primers used for GAPDH were 5' - GTC TCC TCT GAC TTC AAC AGC G -3' and 5'- ACC ACC CTG TTG CTG TAG CCA A -3'. The relative LAIR-1 mRNA expression levels were normalized against GAPDH using the comparative ΔΔCt method and relative fold change of gene was calculated by the equation 2^-ΔΔCt^.

Protein level was detected by Western blotting analysis. Cells were lysed using RIPA lysis buffer (Beyotime, China). Equal amounts of protein were separated by SDS-PAGE and transferred to PVDF membrane, blocked with 5% skim milk. The membrane was probed with a monoclonal mouse anti-LAIR1 antibody [lc12] (1: 500) (ab14826) followed by HRP–conjugated goat anti-mouse antibody (1: 6000) incubation. Protein bands were detected by an ECL kit (Pierce, USA) according to the manufacturer's instructions. The immunoblot of GAPDH was used as a loading control.

### Chromatin immunoprecipitation (CHIP)

Jurkat and COC1 cells were used for CHIP experiments. The proteins were cross-linked to the DNA by treating cells with 1% formaldehyde for 10 minutes. The cross-linking reaction was then terminated by the addition of glycine to a final concentration of 0.125 M. The cells were lysed, and cross-linked chromatin was sonicated using a Biorupter (Diagenode) to generate DNA-fragments of approximately 200 to 1000 bp in length. Anti-ETS-2, which was used to precipitate the chromatin fragments were pre-bound to Biomag^TM^ magnetic beads (Bangs laboratories. Inc). The beads were added to the cell lysates and incubated overnight. The chromatin-antibody complex was then precipitated using a One-Day Chromatin immunoprecipitation kit (Millipore) and digested with proteinase K and RNase to remove the proteins and RNAs. The CHIP DNA was analyzed using PCR amplification. The primers flanking the LAIR-1 gene core promoter were: 5’-ACG GTC ATC TGC CTC TAA GC-3’ (forward) and 5’-CAC CAA TGC AAG GAC AGA ACT C-3’ (reverse). The resultant PCR products were analyzed by 2% agarose gel electrophoresis.

### Electrophoretic mobility shift assays (EMSA)

Nuclear extracts were prepared from Hela cells using a nuclear protein preparation kit (Viagene Biotech Co., Ningbo, China). Hela cells were used because of high expression of ETS-2 [23]. EMSA assays were performed using the Complete Non-radioactive EMSA kit (Viagene Biotech Co., Ningbo, China), according to the manufacturer’s instructions and a biotin end-labeled DNA duplex of sequence containing ETS-2 putative binding site A (5’- TCT CAC TAG TGG GAA GGG TGG AAA GAA CAG-3’; 5’-CTG TTC TTT CCA CCC TTC CCA CTA GTG AGA-3’ ), site B (5’-GGA CAG AAA GCT CTT CCT CTT GTG TGA GGC-5’; 5’-GCC TCA CAC AAG AGG AAG AGC TTT CTG TCC-3’) or site C (5’-CGG GCA GCA CAT CCT GTC TGC TTG TGT c-3’; 5’-GAC ACA AGC AGA CAG GAT GTG CTG CCC-3’). Briefly, nuclear protein samples were incubated for 15 minutes at 4°C with 1.0 μg/μL poly (dI-dC) and biotin-labeled and non-labeled competitor oligonucleotide probes in 10× binding buffer. The resulting DNA-protein complex was separated from free oligonucleotide on a 6% polyacrylamide gel, and the samples were then electrotransferred to a positively charged nylon membrane. The membrane was immediately cross-linked for 15 min on a UV transilluminator equipped with 312 nm bulbs. A chemiluminesce method that utilized a luminol/enhancer solution and a stable peroxide solution was used for detection, and the membranes were exposed to x-ray films for 2-5 min before development.
